# Life without glutamate: the epigenetic effects of glutamate deletion

**DOI:** 10.3389/fnmol.2014.00014

**Published:** 2014-02-26

**Authors:** Lawrence Edelstein, Kjell Fuxe, John Smythies

**Affiliations:** ^1^Medimark CorporationDel Mar, CA, USA; ^2^Department of Neuroscience, Karolinska InstitutetStockholm, Sweden; ^3^Department of Psychology, Center for Brain and Cognition, University of California at San DiegoLa Jolla, CA, USA

**Keywords:** glutamate, epigenetics, exosomes, homeoproteins, Otx2, microRNAs

## Introduction

Li et al. (2013) studied ThVGdKO mice produced by genetic elimination of the genes for both vesicular transporters Vglut-1 and Vglut-2. In these mice thalamocortical glutamate neurotransmission in somatosensory cortex (but not in visual or auditory cortex) is silenced or knocked-out, thereby disabling the release of glutamate from synaptic vesicles. This resulted in a failure to construct barrel cortex columns, and induced severe defects in both cortical layer 4 lamination and in the morphology of spiny stellate cells. The authors explain this finding as follows: “We favor a model in which stellate neurons modulate activity and direct the local migration of neurons into barrels, modify gene expression, and influence cell morphologic development.” Li et al. (2013) present an hypothesis to explain their results: see also the preview of this paper by Barth and Kuhlman (2013). They suggest that it is possible that, by binding to its NMDA or metabotropic receptors, glutamate would stimulate second messenger chains that terminate by activating the appropriate transcription factors. We accept their hypothesis as a partial explanation, but we suggest that there may be other factors involved that they have not considered. To explain these more fully we add two additional hypotheses. These hypotheses are not competitive, either with each other or with the original hypothesis of Li et al. (2013), but constitute additions.

## Hypothesis 1. the role of exosomes

The first factor is the exosome. There is now abundant evidence that almost all cells, neurons included, bud-off exosomes that are, in essence, cargo-carrying vesicles. Exosomes transport genetic material between cells, including protein transcription factors, various types of RNA (including microRNAs), and segments of DNA that may induce phenotypic changes in recipient neurons (Ratajczak et al., 2006; Valadi et al., 2007; Lee et al., 2012; O'Loughlin et al., 2012; Tetta et al., 2012; Turola et al., 2012). The INSERM research group at the University of Grenoble (Fauré et al., 2006; Lachenal et al., 2011; Chivet et al., 2012) has suggested that exosomes may have a regulatory function at synapses and thus may offer an ideal mechanism for the interneuronal transfer of information. Smalheiser (2007) has suggested that exosomes are greatly involved with trans synaptic activity, and that exosomal secretion of proteins and RNAs may play a fundamental role in communication within the nervous system.

These researchers limited their hypotheses of the function of the exosome system to local retrograde activity at the synapses on which the exosomes attach (save a brief mention by Von Bartheld et al., 1996). We have suggested that the function of exosomes that cross the synapse may include anterograde activity in the entire postsynaptic neuron and beyond (Smythies and Edelstein, 2013a,b; Okuno et al., 2014). There is evidence that, from the host cytoplasm, a system of carrier molecules delivers exosomes and their cargoes to the rest of the postsynaptic neuron. Tian et al. (2010) isolated exosomes from PC12 cells, labeled them with a lipophilic dye and an amino-reactive fluorophore. The exosomes were then incubated with these PC12 cells. Live-cell microscopy revealed that the exosomes were endocytosed, enclosed in vesicles and transported to the perinuclear region. This transport may have been mediated by the cytoskeleton. Using a specific technique involving terminal deoxynucleotidyl transferase (TDT) polymerase, Waldenström et al. (2012) identified 343 different chromosomal DNA sequences in the microvesicles/exosomes from cultured cardiomyocytes. Microvesicular/exosomal DNA was observed to transfer into target fibroblasts, where exosomes stained for DNA were seen in the fibroblast cytosol and in the nuclei themselves.

There is now considerable evidence to indicate that microRNAs modulate a number of neuronal functions. For example:

spine numbers and synapse formation are controlled in an activity-regulated manner by miR-485 (Cohen et al., 2011).
transcription of the microRNA miP335 is promoted by naturally evoked synaptic activity at the climbing fiber-Purkinje cell synapse in the mouse cerebellar flocculus (Barmack et al., 2010).
neuronal activity regulates spine formation, in part, by increasing miR132 transcription, which in turn activates a Rac1-protein activated kinase (Rac1-Pak) actin remodeling pathway (Impey et al., 2010).
microR-181a activity in primary neurons, induced by dopamine signaling, is a negative posttranscriptional regulator of GluA2 expression (Saba et al., 2012).
utilizing a mouse line with a conditional neuronal deletion of Dgcr8 (a microRNA biogenesis protein predicted to process microRNAs exclusively) Hsu et al. (2012) produced evidence that some microRNAs govern essential aspects of inhibitory transmission and interneuron development in the mammalian nervous system.
Gennebäck et al. (2013) and de Jong et al. (2012) show that particular modes of stimulation of parental cells by platelet-derived growth factor (PDGF and hypoxia respectively) induce changes in the transcriptional contents of secreted exosomes. This offers new mechanisms of modulation of computational activities in neurons by exosomal payloads.
MiRNA-124a is carried by exosomes from neurons to astrocytes and increases levels of excitatory amino acid transporter (EAAT2) therein (Morel et al., 2013).

The designs of most studies of neuronal microRNA have concentrated on the effects of these agents on the processes within the same neuron that produced them. However, the evidence now suggests that it is possible that microRNAs, as well as other signaling agents, may be exported from a presynaptic neuron by synaptic or perisynaptic transfer via the exosome system, to induce extensive structural and functional changes in the entire postsynaptic neuron.

The relevance of exosomes to the question of the mechanism of action of glutamate deletion is that the INSERM research group at the University of Grenoble report that exosomal release is regulated by K+-induced depolarization. Furthermore, exosome release from neurons in culture is “massively increased” by the introduction of GABA antagonists. This increase is inhibited by both AMPA and NMDA antagonists, indicating that exosome secretion is activated by glutamate activity at both the NMDA and AMPA receptors (Frühbeis et al., 2013). Thus, it would appear likely that, in the genetically engineered glutamate-silenced mice of Li et al. (2013), the release of exosomes is severely reduced. This would suspend a major source of epigenetic factors controlling the structure and function of the postsynaptic neuron. Another link between glutamate activity and exosomes is provided by Frühbeis et al. (2013). They showed that activity-dependent release of the neurotransmitter glutamate triggers oligodendroglial exosome secretion mediated by Ca^2+^entry through oligodendroglial NMDA and AMPA receptors. In turn, neurons internalize the released exosomes by endocytosis. This renders support to the neuron.

It is possible that the roamer type of volume transmission can participate, possibly involving shedding vesicles but mainly exosomes (Agnati and Fuxe, in preparation). They likely play a significant role in the current experiments, and the firing can operate in the case of thalamocortical glutamate neurons in this transgenic model, but without release of glutamate since glutamate is no longer stored in the synaptic vesicles in view of the deletion of the vesicular glutamate transporters. Thus, exosomes with their multiple types of biomolecules (proteins, receptors, mRNA, microRNA, transcription factors, etc.) can be released and internalized into cortical neurons and glia. However, the transcriptional and translational actions of these biomolecules are not sufficient to organize the cortical networks when the multiple glutamate subtypes are not activated by glutamate. It may be that under these conditions the exosomes are not effectively internalized and/or that synergy between the actions of the internalized molecules and the glutamate activated intracellular cascades is necessary for the column and cortical organization.

## Hypothesis 2. the role of Otx2

It is possible that another part of the mechanism responsible may be a homeoprotein such as Otx2 that is carried from the thalamus to the cortex, via the glutamatergic input (Sugiyama et al., 2008; Beurdeley et al., 2012; Carlier et al., 2013; Spatazza et al., 2013). After eye opening, Otx2 starts to be synthesized by the bipolar cells of the retina and is then exported up the visual pathway via retinal ganglion cells, the optic nerve, LGN and optic radiations to target parvalbumin (PV)-expressing GABAergic interneurons (PV cells) in the primary visual cortex. When a critical concentration of Otx2 is reached, plasticity develops in that part of the cortex in order that the circuitry necessary for binocular vision can be established. All during this period, Otx2 is exported from the eye to the cortex, with levels continuing to rise. After a period of time (20 days in mice), Otx2 levels reach another threshold, and plasticity is switched off in order that the cortex can maintain its adult circuitry. However, this maintenance requires continuous transfer of Otx2 from the eye to the cortex. If it is cut-off by deafferentation, plasticity returns.

The activity of Otx2, and of possible related molecules in other modalities of sensory cortex, depends on the continuous activity of the glutamatergic neuron. Therefore, in the absence of glutamate, the supply of homeoprotein may be disrupted. Moreover, in the experiments reported by Li et al. (2013), the glutamate system was never active. Thus, the homeoprotein required for constructing and maintaining PV-expressing GABAergic interneurons was never available. This may well have contributed to the disorganization of the barrels, layer 4 lamination and morphologic reconfiguration observed. Indeed, it is entirely possible that all three mechanism are involved. Further experiments are needed to explore this matter further.

Otx2 may still be released and internalized by the surrounding neurons and glia in this transgenic model, but cannot induce its transcriptional actions and regulation of local protein synthesis in the absence of continuously released glutamate activating multiple glutamate receptor subtypes with their signaling cascades to the nucleus.

It may be proposed that the hypothetical homeoprotein *inter alia* increases the synthesis of certain types of receptor tyrosine kinases (RTKs), which can increase the signaling of discrete trophic factors like fibroblast-derived growth factor (FGF2) or brain-derived neurotrophic factor (BDNF). However, to obtain the necessary synergistic activation to produce the barrel and lamina organization of the cortex, it may be necessary to form *inter alia* receptor tyrosine kinase (RTK)-NMDAR and RTK-classI mGluR heteroreceptor complexes in the plasma membrane, which may demand continuously activated glutamate receptors (Borroto-Escuela et al., 2013). Such molecular events may help lead to the epigenetic changes necessary to produce the barrels, etc. and the exosomally derived biomolecules may contribute when the above circuits are in place.

## References

[B2] Barmack N. H. Qian Z. Yakhnitsa V. (2010). Climbing fibers induce microRNA transcription in cerebellar Purkinje cells. Neuroscience 171, 655–665 10.1016/j.neuroscience.2010.09.039 20875844 PMC2989794

[B3] Barth A. L. Kuhlman S. J. (2013). The many layers of specification and plasticity in the neocortex. Neuron 79, 829–831 10.1016/j.neuron.2013.08.021 24011997

[B5] Beurdeley M. Spatazza J. Lee H. H. Sugiyama S. Bernard C. Di Nardo A. A. (2012). Otx2 binding to perineuronal nets persistently regulates plasticity in the mature visual cortex. J. Neurosci. 32, 9429–9437 10.1523/JNEUROSCI.0394-12.2012 22764251 PMC3419577

[B6] Borroto-Escuela D. O. Flajolet M. Agnati L. F. Greengard P. Fuxe K. (2013). Bioluminescence resonance energy transfer methods to study G protein-coupled receptor-receptor tyrosine kinase heteroreceptor complexes. Methods Cell Biol. 117, 141–164 10.1016/B978-0-12-408143-7.00008-6 24143976 PMC3921556

[B7] Carlier L. Balayssac S. Cantrelle F.-X. Khemtémourian L. Chassaing G. Joliot A. (2013). Investigation of homeodomain membrane translocation properties: insights from the structure determination of Engrailed-2 homeodomain in aqueous and membrane-mimetic environments. Biophys. J. 105, 667–678 10.1016/j.bpj.2013.06.024 23931315 PMC3738247

[B8] Chivet M. Hemming F. Pernet-Gallay K. Fraboulet S. Sadoul R. (2012). Emerging role of neuronal exosomes in the central nervous system. Front. Physiol. 3:145 10.3389/fphys.2012.00145 22654762 PMC3361079

[B9] Cohen J. E. Lee P. R. Chen S. Fields R. D. (2011). MicroRNA regulation of homeostatic synaptic plasticity. Proc. Natl. Acad. Sci. U.S.A. 108, 11650–11661 10.1073/pnas.1017576108 21697510 PMC3136313

[B11] de Jong O. G. Verhaar M. C. Chen Y. Vader P. Gremmels H. Posthuma G. (2012). Cellular stress conditions are reflected in the protein and RNA content of endothelial cell-derived exosomes. J. Extracell. Vesicles 16, 1 10.3402/jev.v1i0.18396 24009886 PMC3760650

[B14] Fauré J. Lachenal G. Court M. Hirrlinger J. Chatellard-Causse C. Blot B. (2006). Exosomes are released by cultured cortical neurons. Mol. Cell. Neurosci. 31, 642–648 10.1016/j.mcn.2005.12.003 16446100

[B15] Frühbeis C. Fröhlich D. Kuo W. P. Amphornrat J. Thilemann S. Saab A. S. (2013). Neurotransmitter-triggered transfer of exosomes mediates oligodendrocyte-neuron communication. PLoS Biol. 11:e1001604 10.1371/journal.pbio.1001604 23874151 PMC3706306

[B16] Gennebäck N. Hellman U. Malm L. Larsson G. Ronquist G. Waldenström A. (2013). Growth factor stimulation of cardiomyocytes induces changes in the transcriptional contents of secreted exosomes. J. Extracell. Vesicles 17, 2 10.3402/jev.v2i0.20167 24009898 PMC3760655

[B17] Hsu R. Schofield C. M. Dela Cruz C. G. Jones-Davis D. M. Blelloch R. Ullian E. M. (2012) Loss of microRNAs in pyramidal neurons leads to specific changes in inhibitory synaptic transmission in the prefrontal cortex. Mol. Cell. Neurosci. 50, 283–292 10.1016/j.mcn.2012.06.002 22728723 PMC3613865

[B18] Impey S. Davarra M. Lesia A. Fortin D. Ando H. Varlamova O. (2010). An activity-induced microRNA controls dendritic spine formation by regulating Rac1-PAK signaling. Mol. Cell. Neurosci. 43, 146–156 10.1016/j.mcn.2009.10.005 19850129 PMC2818337

[B19] Lachenal G. Pernet-Gallay K. Chivet M. Hemming F. J. Belly A. Bodon G. (2011). Release of exosomes from differentiated neurons and its regulation by synaptic glutamatergic activity. Mol. Cell. Neurosci. 46, 409–418 10.1016/j.mcn.2010.11.004 21111824

[B20] Lee Y. Andaloussi S. Wood M. J. (2012). Exosomes and microvesicles: extracellular vesicles for genetic information transfer and gene therapy. Hum. Mol. Genet. 21, R125–R134 10.1093/hmg/dds317 22872698

[B21] Li H. Fertuzzinhos S. Mohns E. Hnasko T. S. Verhage M. Edwards R. (2013). Laminar and columnar development of barrel cortex relies on thalamocortical neurotransmissionneuron. 79, 970–986 10.1016/j.neuron.2013.06.043 24012009 PMC3768017

[B23] Morel L. Regan M. Higashimori H. Ng S. K. Esau C. Vidensky S. (2013). Neuronal exosomal miRNA-dependent translational regulation of astroglial glutamate transporter GLT1. J Biol Chem. 288, 7105–7116 Epub 2013 Jan 30 10.1074/jbc.M112.410944 23364798 PMC3591620

[B24] O'Loughlin A. J. Woffindale C. A. Wood M. J. (2012). Exosomes and the emerging field of exosome-based gene therapy. Curr. Gene Ther. 12, 262–274 10.2174/156652312802083594 22856601

[B25] Okuno H. Smythies J. Edelstein L. (2014). Ch. 15: Selected areas for future research on the claustrum, in The Claustrum - Structural, Functional and Clinical Neuroscience, eds Smythies J. R. Edelstein L. R. Ramachandran V. S. (San Diego, CA: Academic Press), 365–376

[B26] Ratajczak J. Wysoczynski M. Hayek F. Janoswska-Wiecsorek A. Ratajczak M. Z. (2006). Membrane-derived microvesicles: important and underappreciated mediators of cell-to-cell communication. Leukemia 20, 1487–1495 10.1038/sj.leu.2404296 16791265

[B27] Saba R. Störchel P. H. Aksoy-Aksel A. Kepura F. Lippi G. Plant T. D. (2012). Dopamine-regulated microRNA MiR-181a controls GluA2 surface expression in hippocampal neurons. Mol. Cell. Biol. 32, 619–632 10.1128/MCB.05896-11 22144581 PMC3266602

[B27a] Smalheiser N. R. (2007). Exosomal transfer of proteins and RNAs at synapses in the nervous system. Biol. Direct. 2:35 10.1186/1745-6150-2-35 18053135 PMC2219957

[B28] Smythies J. Edelstein L. (2013a). Interactions between the spike code and the epigenetic code during information processing in the brain. Front. Mol. Neurosci. 6:17 10.3389/fnmol.2013.00017 23847467 PMC3703540

[B29] Smythies J. Edelstein L. (2013b) Transsynaptic modality codes in the brain: possible involvement of synchronized spike timing, microRNAs, exosomes and epigenetic processes. Front. Integr. Neurosci. 6:126 10.3389/fnint.2012.00126 23316146 PMC3539687

[B30] Spatazza J. Di Lullo E. Joliot A. Dupont E. Moya K. L. Prochiantz A. (2013). Homeoprotein signaling in development, health, and disease: a shaking of dogmas offers challenges and promises from bench to bed. Pharmacol. Rev. 65, 90–104 10.1124/pr.112.006577 23300132

[B31] Sugiyama S. Di Nardo A. A. Aizawa S. Matsuo I. Volovich M. Prochiantz A. (2008). Experience-dependent transfer of Otx2 homeoprotein into the visual cortex activates postnatal plasticity. Cell 134, 508–520 10.1016/j.cell.2008.05.054 18692473

[B32] Tetta C. Ghigo E. Silengo L. Deregibus M. C. Camussi G. (2012). Extracellular vesicles as an emerging mechanism of cell-to-cell communication. Endocrine 44, 11–19 10.1007/s12020-012-9839-0 23203002 PMC3726927

[B32a] Tian T. Wang Y. Wang H. Zhu Z. Xiao Z. (2010). Visualizing of the cellular uptake and intracellular trafficking of exosomes by live-cell microscopy. J. Cell. Biochem. 111, 488–496 10.1002/jcb.22733 20533300

[B33] Turola E. Furlan R. Bianco F. Matteoli M. Verderio C. (2012). Microglial microvesicle secretion and intercellular signaling. Front. Physiol. 3:149 10.3389/fphys.2012.00149 22661954 PMC3357554

[B34] Valadi H. Ekstrom K. Bossios A. Sjostrand M. Lee J. J. Lotvall J. O. (2007). Exosome-mediated transfer of mRNAs and microRNAs is a novel mechanism of genetic exchange between cells. Nat. Cell Biol. 9, 654–659 10.1038/ncb1596 17486113

[B35] Von Bartheld C. S. Byers M. R. Williams R. Bothwell M. (1996). Anterograde transport of neurotrophins and axodendritic transfer in the developing visual system. Nature 379, 830–833 10.1038/379830a0 8587607

[B36] Waldenström A. Gennebäck N. Hellman U. Ronquist G. (2012). Cardiomyocyte microvesicles contain DNA/RNA and convey biological messages to target cells. PLoS ONE 7:e34653 10.1371/journal.pone.0034653 22506041 PMC3323564

